# Reduced Visual Function in Schizotypal Traits: An Exploratory Study

**DOI:** 10.1093/schbul/sbae049

**Published:** 2025-03-04

**Authors:** Emsal Llapashtica, John L Barbur, Corinna Haenschel

**Affiliations:** Department of Psychology, City, University of London, London, UK; The Henry Wellcome Laboratories for Vision Science, Centre for Applied Vision Research, School of Health and Psychological Sciences, City, University of London, London, UK; Department of Psychology, City, University of London, London, UK

**Keywords:** schizotypy, visual acuity, anomalous perceptions, eye movement responses

## Abstract

**Background and Hypothesis:**

Visual impairments have been proposed as risk factors for psychotic symptoms and illnesses. Visual impairments can considerably impact people’s daily lives, but little is known about the impact and diagnostic sensitivity of such abnormalities for schizotypal personality traits. This study aims to explore possible relationships between schizotypy and visual acuity (VA), contrast sensitivity, and parameters that describe eye movements and visual processing times.

**Study Design:**

Schizotypy was assessed in 37 participants with the Multidimensional Schizotypy Scale-Brief (MSS-B). For the visual function measures, we used the Acuity-Plus test and the new Eye Movement and Integrated Saccade Latency (EMAIL) test. The latter measures oculomotor performance during an eye movement task, including the visual processing time at the end of each saccade.

**Study Results:**

The disorganized dimension of the schizotypy scores predicted VA when measured with black optotypes. Additionally, we found that participants who had higher disorganized scores showed an increased response variability, as assessed through the goodness of fit measure from the EMAIL test.

**Conclusions:**

These results from this exploratory study extend upon earlier findings from both general and patient samples, highlighting the clinical and subclinical importance of understanding how spatial vision can be affected in people with schizotypal disorganized behavior.

## Introduction

Distorted perceptual experience is a well-known and frequently encountered symptom in schizophrenia.^1–3^ It is recognized that these are not restricted to schizophrenia but may occur in as many as 27% of the general population.^4^ When examining the general population, this can be assessed using the personality trait of schizotypy.

Schizotypy incorporates aspects of the three dimensions of schizophrenia: positive, negative, and disorganized. The positive schizotypy dimension includes perceptual alterations and unusual thoughts that bear a resemblance, albeit in a less severe form, to the delusions, and hallucinations of psychosis. Negative schizotypy describes the loss of interest in interpersonal interaction and volition including anhedonia. Lastly, the disorganized dimension represents features that involve odd speech as well as cognitive slippage and odd behavior.^5,6^ Schizotypy exists on a continuum, from a mild subclinical expression in the general population to the full clinical disorder^7,8^ and overlaps with schizophrenia in many behavioral and neurobiological domains.^9^ Longitudinal studies have shown that a large majority of individuals that score high on self-reported schizotypy questionnaires will never experience the full clinical disorder.^10,11^ However, individuals with high levels of schizotypy traits demonstrate some resemblance of milder schizophrenia-like behavior.^9,12^ It is well established that neurophysiological deficits and abnormalities in information processing are common not only at a cognitive^13–17^ but also at the perceptual level.^1,15,18,19^ Diminished visual acuity (VA),^20,21^ abnormalities in contrast sensitivity,^2,22,23^ backward masking,^24,25^ and motion perception^26,27^ are among the most commonly reported early-stage visual processing deficits in schizophrenia patients.^3^ Visual dysfunctions have been shown in people with a clinical high-risk state of psychosis as well as in recent onset psychosis.^28^ Importantly, visual perceptual deficits such as impaired VA can predict the later conversion to psychosis in both high-risk and general populations.^21,29^ A recent longitudinal study in a cohort of 1 million people has demonstrated that impaired VA in late adolescence is associated with non-affective psychosis.^30^

In addition to visual function deficits, there are also reports of oculomotor dysfunction in relation to schizophrenia and schizotypy.^31–35^ Oculomotor tasks have been used in the general population as well as in both schizophrenia and schizotypy to assess externally triggered automatic (reflexive) and internally initiated voluntary eye movement responses.^36–38^ Reflexive prosaccades appear to be relatively unaffected in both schizophrenia and schizotypy^37,39–41^; however, two studies have reported a negative correlation between schizotypy and performance on a prosaccade task.^42,43^

In contrast, eye movement deficits particularly during anti-saccade and smooth pursuit tasks are well documented in both populations.^34,38^ A recent exploratory study also suggested a link between smooth pursuit eye movement and motion perception in high but not low schizotypy.^35^ One measure that has not, to our knowledge, been examined in relation to schizotypy scores is that of participants’ overall performance during the eye movement task. Commonly, the eye movement tasks measure saccade parameters such as latency, its duration, and accuracy, and in the case of anti-saccade tasks the failure to inhibit reflexive saccades. Here we measured VA, and contrast sensitivity and used the EMAIL test—a new psychophysical technique designed to measure the overall time (Integrated Saccade Latency [ISL]) that participants require to detect the peripheral target (T1), generate the appropriate eye movement (T2), and process a specific stimulus attribute at the end of saccade (T3). We used the Multidimensional Schizotypy Scale-Brief (MSS-B^44^) to measure schizotypy.

Our first aim was to explore the potential link between schizotypy and visual function measures, expecting higher schizotypy scores to correlate with both elevated VA, and contrast sensitivity thresholds. The second aim was to explore the relationship between schizotypy and a number of oculomotor parameters contributing to the ISL measured with the EMAIL test.

## Methods

### Participants

Participants were recruited via Sona, which was made accessible to the general public. Exclusion criteria included: age 18 or above and having normal or corrected-to-normal vision. Forty-two participants were recruited. Five participants failed to correctly complete the questionnaires and/or had missing vision tests and were excluded. The remaining 37 (12 males and 25 females, age range: 18–55 years; mean = 29.25 [SD 11.26]) were included in the analysis. Written consent was obtained from all participants. The study had ethical approval from the Department of Psychology Research Ethic Committee, City, University of London.

### Psychometric Assessment: Questionnaires

Schizotypal traits were measured using the 38-item Multidimensional Schizotypy Scale-Brief (MSS-B^44^). The MSS-B consists of 38 items (13 positive, 13 negative, and 12 disorganized items). A score for each dimension and the overall score were produced for each participant. Gross et al (2018) reported Cronbach’s alpha for positives (α = .80), negative (α = .80), and disorganized (α = .90). This was confirmed here with Cronbach’s alpha for positive α = .66, negative α = .81, disorganized α = 0.87. While the focus of the paper was on schizotypy, we also measured psychotic-like experiences (PLEs) using the Cardiff Anomalous Perceptions Scale (CAPS), and the Peters et al. Delusions Inventory (PDI). Following a comment by one of the reviewers, this was dropped from the main analysis. Non-significant results are shown in the Supplementary material.

### Procedure

Participants completed the questionnaires online via Qualtrics and were invited to carry out two psychophysical tests: (1) The Acuity-*Plus* test, measures both VA and functional contrast sensitivity (FCS) thresholds using both positive (white) and negative (black) contrast optotypes. (2) Oculomotor responses were assessed using the Eye Movements and Integrated Saccade Latency (EMAIL) test. The experiments were carried out in a darkened room. Participants used a chin rest to view the visual display from a distance of 300 cm for the Acuity-Plus test and 80 cm for the EMAIL test. The uniform background field had a luminance of 32 cd/m^2^ and CIE (*x*, *y*) chromaticity coordinates of 0.305, 0.323 (which approximates daylight at 6500K). Each participant completed all threshold measurements in a single session. Participants were encouraged to take short breaks between the tests to minimize fatigue.

### Acuity-Plus: Procedure

**Fig. 1.**
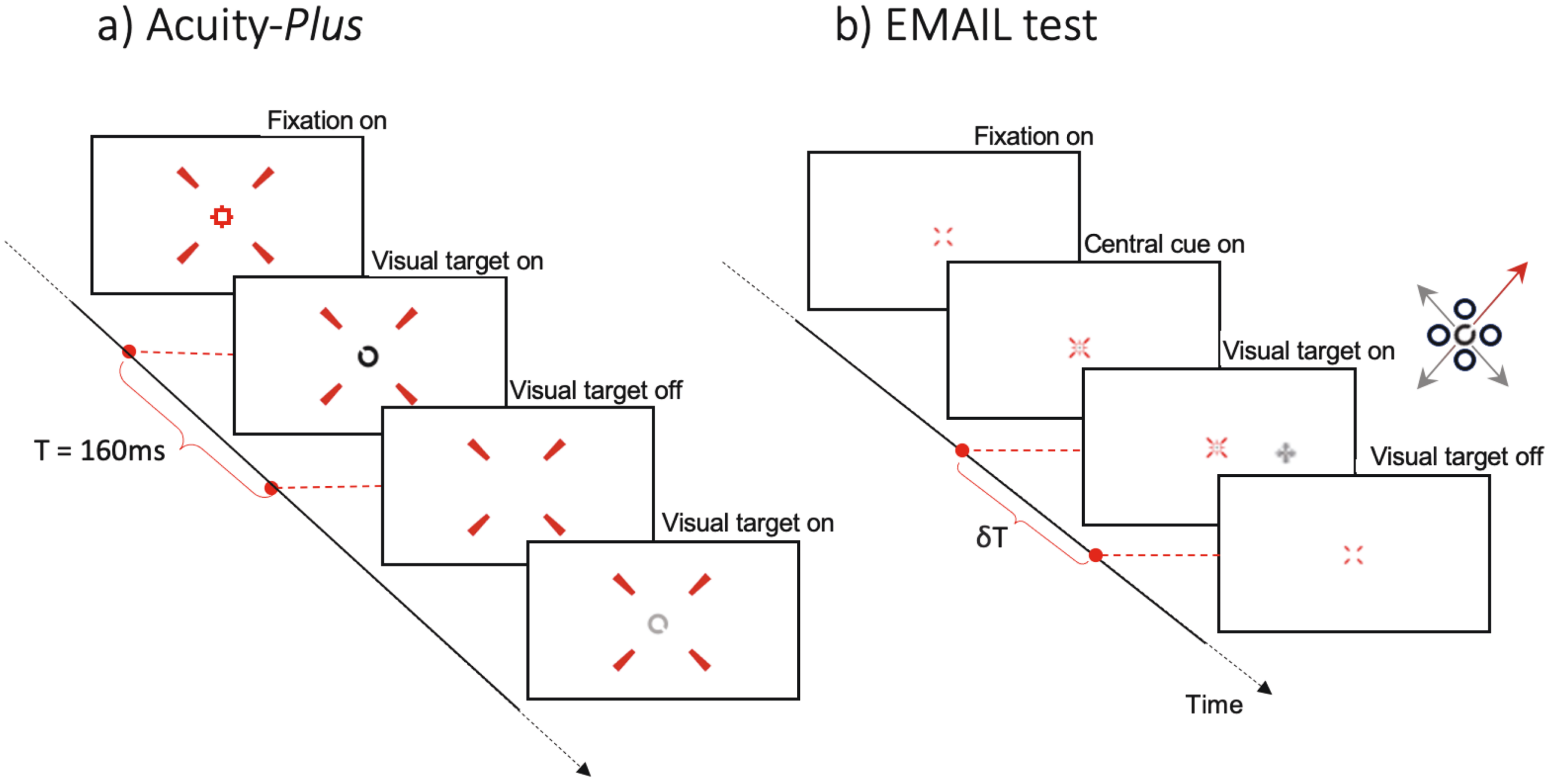
Schematic representation of the timeline employed in the (a) Acuity-*Plus* test and (b) EMAIL test. In both tests, the participant’s task is to detect and register the orientation of the gap in the Landolt C optotype. EMAIL, Eye Movement and Integrated Saccade Latency.

A four-alternative, forced-choice (4AFC) staircase procedure with variable step sizes was used to measure VA threshold and CS. The threshold measured corresponds to 71% probability of correct response. The test target consisted of a Landolt C optotype with the gap positioned randomly in one of four randomly interleaved diagonal directions. The participant’s task was to detect and “register” the orientation of the gap in the Landolt C (see figure 1a for timeline). VA and CS thresholds were measured with both positive (white) and negative (black) contrast optotypes (for more details for the Acuity-Plus test^45^). Best-corrected-visual-acuity (BCVA) values for the stimulus employed in this study range from 0.5 to 1.25 min arc. Using previously established age-normed medians for each participant’s VA thresholds we calculated the mean median and the mean upper normed VA threshold limit^45^ (UNL). Additionally, we computed the difference between each participant’s age-normed median and their actual score.

### EMAIL Test: Procedure

A 4AFC staircase procedure with variable step sizes was used to measure ISL. The staircase varied the stimulus presentation time, using a “2-down, 1-up” procedure to achieve a 71% correct response rate. The test employed an overlap paradigm to trigger visually guided saccades (see figure 1b). The test target consisted of a Landolt C with a gap size of four arc minutes surrounded by four ring distractors of similar size. During each stimulus presentation, the position of the gap in the Landolt C was selected randomly to correspond to one of the four diagonal directions, as shown in figure 1b. The participant was required to saccade to the stimulus and to “register” the orientation of the gap in the Landolt ring via button press (figure 1b).

We ran two experimental sequences to measure the participants’ thresholds for ISL times. First, we measured the participant’s ISL values for 75% stimulus contrast presented peripherally at an eccentricity of 8°—randomly on either side of fixation. The subject-specific thresholds ranged between 174 and 262 ms. Second, we repeated the above measurement, but for fixed stimulus presentation times selected to fall within 20 ms, both above and below the participant’s measured ISL time, and recorded the eye movements. For each run the stimulus was presented at least 50 times in order to estimate the time course of the saccade and the probability of a correct response. For the combined EMAIL/Eye-tracker experiments a custom-made photodiode attached to a corner of the display measured accurately the actual stimulus time on the display and determined the probability of making a correct response for each discrete stimulus presentation time.

### Eye Movement Recordings and Analysis

Eye movements were measured using the EyeLink 1000 eye-tracker at a sampling frequency of 1000 Hz. The experiments were performed binocularly, but the eye movement traces were measured only in the right eye. Each trial began with an EyeLink, 9-point calibration routine, and was followed by a validation check to evaluate the gaze accuracy. Three successive test runs were needed to complete the experiment. For the first trial, the stimulus duration corresponded to the participants’ ISL time measured with the EMAIL test, whereas during the second and third runs, stimulus durations were both above and below the measured ISL time. A Weibull function was then fitted to the measured data in order to estimate the time the participant required to achieve the probability of 71% correct response (ie, the ISL time). For the second experiment, a custom-made algorithm was used to detect saccades from eye movement recordings and estimate the following parameters that make up the ISL thresholds. The saccadic latency (T1) represents the time from the onset of the target to the initiation of the saccade, whereas the difference between saccade offset and onset time defined the duration of the eye movement (T2) and T1 + T2 determined the end of the saccade. T3 was estimated by subtracting T1 + T2 from ISL and represents the remaining stimulus time the subject can use to process the visual stimulus after the saccade has ended. All trials with blinks and anticipatory latencies shorter than 60 ms were excluded. Only correct responses were included in the final analysis. These were adjusted for chance probability prior to fitting the data with the Weibull function to obtain participants’ ISL thresholds. Generally, the slope of the psychometric function determines both the participants’ level of response and/or the variability of the responses. As such, the slope can be used to tell whether the difference is caused by reduced response or increased variability. To determine variability, goodness of fit (GOF) was used as a statistical measure to describe how well the participant’s data fitted the function.

### Statistical Analysis

We used stem and leaf plots to detect extreme outliers in the visual measures. An extreme outlier was identified for VA^−^ threshold, but not for the other measures. To limit the influence of this outlier on our statistical analysis we applied winsorization^46^ and replaced the value with the next largest value.

We run three separate zero-order Pearson correlations for: (1) VA threshold, (2) contrast sensitivity, and (3) saccadic variables (T1, T2, T1 + T2, T3, and ISL) with the positive, negative, and disorganized schizotypy score to guide the regression analysis. We used false discovery rate (FDR) correction^47^ to correct for multiple comparisons. To avoid interpreting small effects, we considered effects as statistically significant only when their effect sizes were ≤30 (as determined by Cohen’s *d*). Significant effects with medium to high effect sizes were investigated using multiple regression analysis to assess the combined effects of schizotypy on visual/saccadic outcome measures. This was followed up with robust regression to confirm that results were not due to skewed data and to validate the reliability of the predictors. We confirmed that the residuals were normally distributed by calculating the *Q*–*Q* plots before running the regression analysis. All the data were analyzed using MATLAB and SPSS software and R for the robust regression (see Supplementary material).

## Results

Descriptive statistics of visual function measures, saccadic variables, T1–T3, ISL, and psychometric questionnaires are given in tables 1 and 2, respectively.

**Table 1.** Descriptive Statistics of CS and VA Threshold Thresholds for Both Positive and Negative Polarity, Oculomotor Variables, Visual Processing Times, and Integrated Saccade Latency (ISL) Values

	Mean	SD
Acuity-Plus		
Contrast Sensitivity (CS^+^)—positive polarity (%)	17.26	14.36
Contrast Sensitivity (CS^−^)—negative polarity (%)	17.32	14.38
Visual Acuity (VA threshold)—positive polarity (arcmin)	1.34	0.53
VA + threshold Normed Median (UNL)	0.91 (1.52)	0.05
Visual Acuity (VA threshold)—negative polarity (arcmin)	1.27	0.48
VA^−^ Threshold Normed Median (UNL)	0.84 (1.43)	0.06
EMAIL test		
Saccadic latency, T1 (ms)	123.4	12.64
Saccadic duration, T2 (ms)	40.4	3.42
End of saccade, T1 + T2 (ms)	163.7	13.32
Integrated saccade latency, ISL = (T1 + T2 + T3) ms	215.6	20.0
Visual processing time (ms), T3 = (ISL − (T1 − T2)) ms	52	12.5

*Note*: Please note VA threshold means are within the upper normed limit.^44^

EMAIL, Eye Movement and Integrated Saccade Latency; UNL, upper normed limit.

**Table 2.** Means, Standard Deviations, Minimum, Maximum, for the 38-Item MSS-B, Multidimensional Schizotypy Scale-Brief

MSS-B	Mean	SD	Range
Positive	2	2.5	0–9
Negative	2.4	2.1	0–8
Disorganized	2.3	3	0–12
Overall	6.6	5.1	0–25

*Note*: MAA-B, Multidimensional Schizotypy Scale-Brief.

### Visual Acuity

There was a significant positive correlation between participants’ disorganized scores and VA^−^ thresholds (95% CI 0.13–0.65), as well as the difference in median score (95% CI 0.14–0.67) (table 3). The FDR-corrected adjusted *P*-value was .01.

**Table 3.** Pearson Correlations Between the Schizotypy Dimensions and VA^−^ Threshold, the Difference From the Age-normed Median (Median Diff) and Saccadic Variables Including the Psychometric Curve Fit GOF

	VA^−^ Threshold	Median Diff	T1	T1 + T2	T3	ISL	GOF
MSSB							
Positive	0.08	0.12	−0.1	−0.08	−0.02	−0.07	−0.06
Negative	0.26	0.23	−0.09	−0.13	−0.09	−0.15	−0.16
Disorganized	**0.41** **	**0.44** **	−0.37*	**−0.40** **	0.19	−0.15	−0.43**

*Note*: GOF, goodness of fit; VA, visual acuity.

Variables that presented corrected significant effect at *P* < .01 are underlined. Medium effect sizes are in bold (*r* effects: medium ≥ .30). Correlation is in trend significant at a level of

*P ≤ .05;

**Correlations significant at a level of *P* ≤ .01

### Contrast Sensitivity

We then calculated the correlations between CS^+^ and CS^−^ and schizotypy dimensions (FDR corrected *P*-value was .008), but there were no significant correlations with schizotypy scores.

### Eye Movement and Integrated Saccade Latency

We found an uncorrected significant negative correlation between disorganized and the GOF (95% CI −0.66 to −0.12), (see table 3; FDR corrected *P*-value was .003). Additionally, the results indicated an uncorrected significant negative correlation between disorganized and latency (T1) (95% CI −0.62 to −0.05) and saccade end (T1 + T2) (95% CI −0.64 to −0.09). These were found to decrease with higher scores in the disorganized dimension (see table 3). No other uncorrected significant effects were observed for the parameters measured with the EMAIL test.

**Fig. 2.**
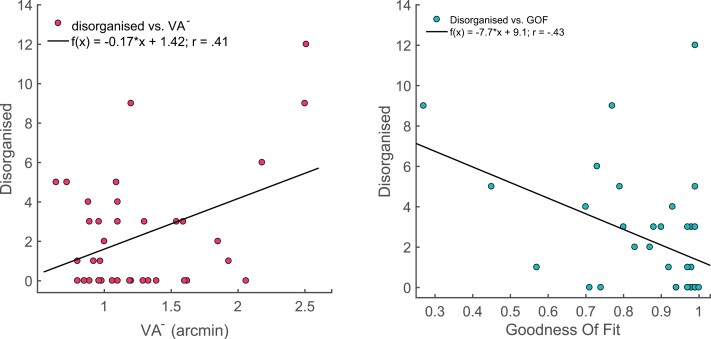
Disorganized scores as a function of VA^−^ threshold and GOF. Each plot shows the observed trend. The correlation coefficient (*r*) value and the corresponding regression equation are shown. GOF, goodness of fit; VA, visual acuity.

To understand the significant effects between the measured visual variables and the dimensions of disorganized, positive, and negative schizotypy, we ran multiple regression analyses (see Figure 2) and robust regression (see Supplementary material). For VA^−^ threshold, the overall model was in trend significant (*F*(3,33) = 2.68, *P* = .06, *R*^2^ = .2) with disorganized schizotypy significantly predicting VA^−^ threshold (β = .06, *P* = .031), while positive and negative schizotypy did not (β = −.004, *P* = .89; β = .037, *P* = .33, respectively). A similar pattern was found for the difference to age-corrected median scores. The overall model was significant (*F*(3,33) = 2.96, *P* = .047, *R*^2^ = .21) with disorganized schizotypy significantly predicting the difference to age-corrected median (β = .68, *P* = .02), whereas positive (β = .002, *P* = .95) and negative (β = .03, *P* = .43) schizotypy were not significant. For GOF, the overall model was significant (*F*(3,33) = 4.07, *P* = .015, *R*^2^ = .27) with disorganized schizotypy negatively predicting the GOF (β** **= −.03, *P* = .002), while positive (β = .007, *P* = .54), and negative schizotypy (β = .023, *P* = .07) had no predictive effect on GOF. In contrast, for both T1 and T1 + T2, the overall model was not significant (*F*(3.33) = 1.77, *P* = .17, *R*^2^ = .14 and *F*(3.33) = 2.17, *P* = .11, *R*^2^ = .17, respectively). However, disorganized schizotypy still predicted T1 and T1 + T2 (β = −1.69, *P* = .04 and β = −1.81, *P* = .03, respectively). This was not the case for positive (β = .05, *P* = .96; β = .16, *P* = .86) and negative schizotypy (β = .007, *P* = .99; β = −1.75, *P* = .87), respectively. These results were confirmed by robust regression except for T1 + T2 that remained significant for the disorganized schizotypy (see table 3 and Supplementary material).

## Discussion

We used multiple regression analysis to explore the degree to which VA and different aspects of visually guided saccades are related to schizotypy. We found a significant association between VA thresholds and the disorganized dimension of schizotypy. In addition, the disorganized dimension showed a negative association with GOF and T1 + T2. Impaired VA is known to be associated with psychosis and has been suggested as a risk for later conversion to schizophrenia.^1,21,30^ Furthermore, there is strong evidence linking visual distortions and diminished VA.^1,21,48,49^ The findings presented here extend this evidence by demonstrating that VA is also associated with the disorganized but not the positive dimension of schizotypy. This remained significant when taking previous age-corrected medians for VA into account.^47^ This observation is in line with previous research showing that disorganization has been shown to be higher in people in individuals at risk^50,51^ and has also been linked to perceptual abnormalities.^52^ Torrens et al^52^ used the pattern glare test (PGT), a test where participants have to report visual illusions and sensations detected in the spatial grating and found that disorganized but not positive schizotypy predicted PGT scores. These results suggest that diminished VA could serve as a relevant biomarker in both schizophrenia and schizotypy. However, it remains unknown whether the mechanisms underlying the VA deficits are similar for both groups. It is well established that in addition to the eye’s optics, the number of photoreceptors as well as various retinal and neural diseases can contribute to diminished VA.^53,54^ Thus, these findings highlight the importance of identifying the specific mechanisms involved. Silverstein et al^54^ suggested that impaired visual functioning in schizophrenia as early as the retina would cause weaker signaling at the subcortical and possibly cortical levels, which might be due to changes in retinal dopamine that would result in a lowered signal-to-noise ratio.^55^ In fact, even small acuity differences within the normal range have been shown to predict the ability to detect and integrate Gabor elements, especially at higher spatial frequencies.^56^ Hence, it may be the case that small differences in VA in schizotypy may have a similar effect on perceptual and cognitive performance.

The second aim was to explore the relationship between schizotypy and a number of oculomotor parameters contributing to the integrated saccade latency (ISL) measured with the EMAIL test. The disorganized dimension showed a negative association with GOF and T1 + T2. While we did not observe any significant effects between the domains of schizotypy and visual processing times, including the ISL thresholds, the GOF measure, which determines the participant’s overall performance in relation to the stimulus duration time, revealed a significant negative correlation with the disorganized dimension scores. Participants with higher scores on the disorganized schizotypy dimension showed an increase in response variability.

Poorer performance on the EMAIL test can be related to the disorganized dimension via response inhibition. We observed a trend towards enhanced eye movement responses for T1 and T1 + T2 (table 3), which is indicative of a trade-off between speed and accuracy, as revealed by the GOF measure. The findings between disorganized schizotypy and shorter durations for both T1 and T1 − T2 are consistent with a previous observation.^43^ The regression analysis suggests that the disorganized dimension was associated with a significant effect on T1 + T2 and GOF revealing a change in the GOF within participants. One possible interpretation of GOF is that it reflects increased variability within participants. This would align with previous studies that have shown increased intra-subject reaction time variability for schizophrenia.^57^ In addition, previous studies have also associated the disorganized dimension with attentional deficits.^6^ Thus, poorer attention and reduced accuracy being the underlying features of the disorganized dimension may account for our findings in relation to poorer performance as captured with GOF. Consequently, GOF can be a sensitive measure to detect reaction time variability in the disorganized dimension.

We did not observe any relationship between contrast sensitivity and schizotypy scores. This finding contrasts with a previous study that reported a connection between contrast sensitivity and schizotypy scoresi^58^ (although see^59^ for contrasting results). An explanation for this discrepancy might lie in design differences as Harper et al^58^ presented a grating as well as a moving pattern using different spatial and spatiotemporal contrast sensitivity measures, whereas we presented Landolt C optotypes, as well as the sample size (*N* = 73 vs. *N* = 39).

This is the first exploratory study to assess differences in VA within schizotypy, however, there are several limitations that require a cautious interpretation of the results. A key limitation is the sample size, and as such, the estimates of the association between schizotypy and visual function might be sensitive to sampling variations and extreme values. Another limitation of our exploratory study was the number of variables used to investigate the relationship between vision and schizotypy dimensions. To confirm the specific effects and to reduce the likelihood of an overestimated effect size due to low power and build evidence for a future, further (preregistered) and larger studies are needed to replicate and confirm the findings and build evidence for a future meta-analysis.

Nevertheless, the results of the regression analysis, highlight the potential significance of our findings and emphasize the importance of gaining a better understanding of the connection between these visual measures and schizotypy.

In summary, we have shown that both diminished VA and, to a degree, poorer overall performance on the EMAIL test were associated with disorganized schizotypy dimension scores. These findings suggest a link between VA and schizotypy, aligning with existing evidence indicating that VA is associated with an increased risk of later conversion to schizophrenia. These results suggest that visual abnormalities may be a risk factor for the development of schizotypy and more broadly within the schizophrenia spectrum. This in line with the “Protection against Schizophrenia” model which proposes that aberrant visual input contributes to the development of schizophrenia.^60^

In addition, the absence of a significant correlation with positive contrast (ie, “white” optotypes) on the VA test raises interesting questions about the processing of “white” and “black” stimulus polarities in measures of spatial vision. The immediate question of interest is why VA with “white” optotypes does not show the same correlation with the disorganized dimension scores of the schizotypy (MSS-B) test. The Acuity*-Plus* test employs briefly presented stimuli to avoid eye movements and multiple glimpses. This makes the test different to conventional, chart-based tests of VA. The brief stimulus duration cannot explain the results from this study since the same duration was employed for both contrast polarities. Perceptually, the two stimuli are not, however, equivalent. A “black” optotype of maximum contrast has a well-defined endpoint since the luminance of the optotype cannot be made less than zero. −100% is therefore the largest contrast and the corresponding perceptual experience one can produce. “White” optotypes have no such limit and can therefore be several times brighter than the adjacent background field, resulting in much larger Weber contrast values, with no clearly defined limit on the corresponding perceptual experience.

Future studies are therefore needed to investigate the perceptual differences between “white” and “black” optotypes in relation to schizotypy test scores to establish the reasons for these findings. Equally important, further studies are needed to investigate whether the specific correlation between VA measured with black optotypes and the disorganized dimension of the schizotypy test is clinically relevant.

## Supplementary Material

Supplementary material is available at https://academic.oup.com/schizophreniabulletin/.

sbae049_suppl_Supplementary_Material
