# Supplementary material for: Reduced Visual Function in Schizotypal Traits: An Exploratory Study
Source: Schizophr Bull. 2025 Mar 4;51(Suppl 2):S205–13. doi: 10.1093/schbul/sbae049 (PMC11879504; doi:10.1093/schbul/sbae049)
Supplement: sbae049_suppl_Supplementary_Material [file sbae049_suppl_supplementary_material.docx]

**Supplementary Data**

As part of the larger project, we also explored the associations between psychotic-like experiences (PLEs) and visual functioning. These states were assessed using the Cardiff Anomalous Perceptions Scale (CAPS, Bell et al., 2006) and the Delusions Inventory (PDI, Peters et al., 2004).

The 21 item PDI schizotypal scale is designed as a scale for the measurement of delusional ideation that incorporates measures of distress, preoccupation and conviction. Four separate scores were obtained from PDI for each participant: 1. Total number of items endorsed, 2. Distress score, 3. Preoccupation score and 4. Conviction score. The 32 item CAPS was developed as a measure of perceptual anomalies, rather than typical schizotypal traits. The four subscales of CAPS measure distress, intrusiveness, total number of items endorsed and frequency of anomalous experience.

TABLE 1. Means, standard deviations and range for the 32-item CAPS and the Cardiff Anomalous Perceptions Scale.

|  | **Mean** | **S.D** | **Range** |
| --- | --- | --- | --- |
| CAPS-32 |  |  |  |
| Distress | 12.9 | 17.64 | 0-86 |
| Intrusiveness | 14.97 | 17.57 | 0-78 |
| Frequency | 12.62 | 13.82 | 0-54 |
| Yes endorsed | 5.59 | 5.36 | 0-16 |
| PDI – Distress score | 5.6 | 5 | 0-19 |
| PDI – Preoccupation score | 5.6 | 5 | 0-19 |
| PDI – Conviction score | 5.6 | 5 | 0-19 |
| PDI – Total score | 21.8 | 19.3 | 0-70 |

**TABLE 2**. Pearson correlations between the scores of CAPS-32, PDI-21 and visual acuity and saccadic variables including GOF. The significant r values are presented with asterisk. Note that only variables that presented significant effect at p<.01 are highlighted. (Correlation is in trend significant at level of *P = <.05; **Correlations significant at level of P = <.01**,** FDR corrected adjusted *p*-value for visual acuity was 0.01).

|  |  | VA Threshold | T1 | T1+T2 | T3 | ISL | GOF |
| --- | --- | --- | --- | --- | --- | --- | --- |
| **CAPS** | Distress | *0.38** | -0.14 | -0.13 | 0.12 | -0.2 | -0.1 |
|  | Intrusiveness | 0.31 | -0.13 | 0.14 | 0.17 | 0.01 | -0.22 |
|  | Frequency | 0.25 | -0.17 | -0.19 | 0.13 | -0.05 | -0.25 |
|  | Endorsed | 0.21 | -0.13 | -0.15 | 0.20 | 0.03 | -0.31 |
| **PDI-21** | Distress | 0.13 | -0.12 | -0.13 | 0.06 | -0.05 | -0.23 |
|  | Preoccupation | 0.13 | -0.11 | -0.13 | 0.06 | -0.05 | -0.23 |
|  | Conviction | 0.13 | -0.11 | -0.13 | 0.06 | -0.05 | -0.23 |
|  | Yes score | 0.13 | -0.11 | -0.14 | 0.14 | -0.01 | -0.29 |
|  | Total score | 0.13 | -0.12 | -0.13 | 0.08 | -0.042 | -0.25 |

Bell V, Halligan PW, Ellis HD. The Cardiff Anomalous Perceptions Scale (CAPS): A new validated measure of anomalous perceptual experience. *Schizophr Bull*. 2006;32(2):366-377. doi:10.1093/schbul/sbj014

Peters E, Joseph S, Day S, Garety P. Measuring delusional ideation: The 21-item Peters et al. Delusions Inventory (PDI). *Schizophr Bull*. 2004;30(4):1005-1022. doi:10.1093/oxfordjournals.schbul.a007116

**Table 3**: Robust regression using R for VA-threshold and saccadic variables including the psychometric curve fit GOF, latency T1 and saccade end (T1+T2). Table shows ß.

Robust Regressions using the rlm function

|  | VA threshold | GOF | latencyT1 | T1T2 |
| --- | --- | --- | --- | --- |
| (Intercept) | 1.024*** | 0.930*** | 125.558*** | 167.096*** |
| Positive | -0.007 | -0.005 | -0.019 | 0.049 |
| Negative | 0.041 | 0.014+ | 0.447 | 0.212 |
| Disorganised | **0.060*** | **-0.021***** | -1.516+ | -1.728* |
|  |  |  |  |  |
| + p < 0.1, * p < 0.05, ** p < 0.01, *** p < 0.001 | | | | |

R Studio (R Core Team, 2023) and the default settings of the rlm function (part of the MASS package in R, Venables & Ripley, 2002) was used for the analysis of the robust regression.

R Core Team (2023). R: A Language and Environment for Statistical Computing. R Foundation for Statistical Computing, Vienna, Austria. [https://www.R-project.org/](https://www.r-project.org/).

Venables, W. N. & Ripley, B. D. (2002) Modern Applied Statistics with S. Fourth Edition. Springer, New York. ISBN 0-387-95457-0
